# Reemergence of Ebola virus in Africa.

**DOI:** 10.3201/eid0103.950307

**Published:** 1995

**Authors:** A Sanchez, T G Ksiazek, P E Rollin, C J Peters, S T Nichol, A S Khan, B W Mahy


### Reemergence of Ebola Virus in Africa

Members of the family *Filoviridae*, which currently consists of Ebola and Marburg viruses, cause severe and often fatal hemorrhagic fevers in humans and nonhuman primates. The recent isolation and identification of a new Ebola virus from a single nonfatal human case in Côte d'Ivoire (1) and the more recent outbreak of Ebola hemorrhagic fever in and around Kikwit, Zaire (2, 3), have raised concerns about the public health threat of these human pathogens. Filoviruses are classified as biosafety level 4 agents because of the extreme pathogenicity of certain strains and the lack of a protective vaccine or effective antiviral drug. Moreover, filoviruses are among the most mysterious groups of viruses known because their natural history and reservoirs remain undefined and their pathogenesis is poorly understood.

Ebola virus infections were first recognized in 1976, when simultaneous but separate outbreaks of human disease caused by two distinct virus subtypes erupted in northern Zaire and southern Sudan (4) and resulted in hundreds of deaths. The Zaire subtype of Ebola virus had a higher case-fatality, nearly 90%, while the Sudan subtype had a case-fatality rate of approximately 50%. Before 1995, the last identified outbreak of Ebola disease in Africa occurred in 1979, when the Sudan subtype of Ebola virus infected 34 persons (5). In late 1989, in Reston, Virginia, a novel Ebola virus infected a colony of cynomolgus macaques that had been imported from the Philippines (6). The new virus, named Reston virus, was shown by researchers at the Centers for disease Control and Prevention (CDC) to be antigenically and genetically distinct from the African Ebola viruses, yet despite its high pathogenicity for nonhuman primates, it did not appear to cause disease in humans. Several persons who handled the infected animals developed antibody to Ebola virus but showed no signs of disease; one of these persons was infected while performing an autopsy on an animal that had died of a Reston virus infection. In 1992, a repeat of the 1989 Reston episode occurred in Siena, Italy when macaques were received from the same Philippine exporter; no evidence of a human infection was found (7). The new Ebola virus recently isolated from a patient in the Côte d'Ivoire has been shown to be genetically distinct from previous Ebola isolates (A. Sanchez, unpublished data) and is the first evidence of Ebola virus in West Africa.

Investigations of these outbreaks, as well as of those caused by Marburg viruses, have yet to produce any substantial evidence for the natural reservoir(s) of filoviruses. Filoviruses do not persist in experimentally infected nonhuman primates; therefore, nonhuman primates are likely not the natural reservoir. Like humans, these species probably are infected when direct or indirect contact is made with the natural host.

The recent news of a large Ebola outbreak in Kikwit, Zaire, alarmed a worldwide audience already sensitized by an array of books, magazine articles, television programs, and movies dealing with the danger of Ebola virus disease. The public concern is underscored by the potential for the spread of these viruses to far regions of the world as a result of international commerce and jet travel. The Kikwit outbreak was similar to the original 1976 episode in Zaire, which was centered around the small village of Yambuku some 1000 km to the north (8). As in the 1976 outbreak, secondary transmission of the virus in Kikwit occurred through close personal contact with infectious blood and other body fluids and was facilitated by the lack of modern medical facilities and medical supplies that could protect those giving care to the initially affected patients. The chief difference between the Yambuku episode and this year's outbreak is that Kikwit is a large and densely populated center close to larger cities, such as Kinshasa and Brazzaville, and the potential for communitywide transmission and spread to neighboring areas is greater. Retrospective case surveillance suggests that the index case may have been a charcoal maker that worked in the forest outside Kikwit. Human to human transmission occurred without being recognized until the end of April 1995. Ebola hemorrhagic fever was suspected when nosocomial infections in the surgical teams and the nursing staff followed repeated laparotomies on an infected laboratory technician in Kikwit General Hospital. Specimens were send to CDC through the Tropical Institute of Antwerpen (Belgium). Teams of experts from CDC, the World Health Organization, Belgium, France, South Africa, and Sweden traveled to the region to assist in implementing safe patient care, management, and containment of the Ebola virus outbreak. As of July 1, 1995, 233 deaths had been reported among the 293 cases.

Rapid diagnosis and characterization of Ebola virus was performed at CDC in Atlanta on blood specimens from 14 patients received on May 9. Nine hours after the specimens had been delivered to CDC, Ebola virus antigen and/or antibody to this virus was confirmed in specimens from 13 of the patients. Four hours later, reverse transcriptase-polymerase chain reaction (RT-PCR) assays targeting conserved regions of filovirus polymerase or Ebola virus glycoprotein genes each detected Ebola virus RNA in 12 of the patients. Subsequent analysis of the genetic profile of the virus was especially important to understanding the epidemiology of the Kikwit outbreak. Within 48 hours of receiving the specimens, sequence analysis on the PCR DNA (528 bp) amplified from the glycoprotein gene derived from four different patients showed that the Ebola virus was a Zaire subtype that differed from the original 1976 strain in four bases (<1%). No differences were seen when the polymerase gene PCR products (~350 bp) from those four patients were sequenced, which indicated that they had been infected with the same virus. Three days later, sequence data from expanded analysis of the entire glycoprotein gene were compared with those of the original 1976 Yambuku isolate (9) and showed that the overall difference between these Ebola viruses was less than 1.6%. Such little change in viruses that caused outbreaks of disease at extreme ends of Zaire separated by a span of nearly 19 years, may indicate that the genomes of Ebola viruses (and filoviruses in general) are unusually stable and have evolved to occupy special niches in the wild.

The capability to rapidly diagnose and characterize filovirus infections is critical to the ability of public health professionals to identify and limit the spread of future outbreaks of filovirus disease. A continued commitment to research and modern disease-surveillance programs is necessary to minimize or preclude filovirus outbreaks similar to that in Kikwit. The possibility of outbreaks is increasingly likely given the continued human incursions into the African forests and the vulnerability of large impoverished populations to rapid transmission of disease as a result of inadequate public health services. With the current outbreak under control, CDC and collaborators have begun their efforts to identify the natural reservoir by sending teams of scientists to collect specimens from the area where the putative index patient worked. Attempts to identify the reservoir after outbreaks in 1976 and 1979 were handicapped by the lack of satisfactory diagnostic tools that are critical to detecting small quantities of the virus. However, now that sensitive enzyme immunoassays and PCR assays have been developed for filoviruses, the chances are much better that, if appropriate materials can be collected in the field, the virus can be detected.

In conclusion, we want to alert physicians and public health agencies who encounter persons that have clinical signs and symptoms of hemorrhagic fever disease to the reemergence of Ebola virus. Recommendations for the management of viral hemorrhagic fevers attributable to filoviruses in the United States were recently published in CDC's *Morbidity and Mortality Weekly Report* (1995;44:475-79).

**Anthony Sanchez, Thomas G. Ksiazek, Pierre E. Rollin, Clarence J. Peters, Stuart T. Nichol, Ali S. Khan, and Brian W. J. Mahy**
National Center for Infectious Diseases
Centers for Disease Control and Prevention
Atlanta, Georgia, USA

### References

1. Le Guenno B, Formenty P, Wyers M, Gounon P, Walker F, Boesch C. Isolation and partial characterisation of a new strain of Ebola virus. Lancet 1995;345:1271-4
2. Centers for Disease Control and Prevention. Outbreak of Ebola viral hemorrhagic fever—Zaire, 1995. MMWR 1995;44:381-2.
3. Centers for Disease Control and Prevention. Update: outbreak of ebola viral hemorrhagic fever—Zaire, 1995. MMWR 1995;44:399.
4. Bowen ETW, Platt GS, Lloyd G, Baskerville A, Harris WJ, Vella EC. Viral haemorrhagic fever in southern Sudan and northern Zaire: preliminary studies on the aetiologic agent. Lancet 1977;1:571-3.
5. Baron RC, McCormick JB, Zubeir OA. Ebola virus disease in southern Sudan: hospital dissemination and intrafamilial spread. Bull WHO 1983;62:997-1003.
6. Jahrling RB, Geisbert TW, Dalgard DW, et al. Preliminary report: isolation of Ebola virus from monkeys imported to USA. Lancet 1990;335:502-5.
7. World Health Organization. Viral haemorrhagic fever in imported monkeys. Wkly Epidemiol Rec 1992;67:142-3.
8. World Health Organization. Ebola haemorrhagic fever in Zaire, 1976. Bull WHO 1978;56:271-93.
9. Sanchez A, Kiley MP, Holloway BP, Auperin DD. Sequence analysis of the Ebola virus genome: organization, genetic elements, and comparison with the genome of Marburg virus. Virus Res 1993;29:215-40.

### Prospects for the Control of Bolivian Hemorrhagic Fever

Bolivian hemorrhagic fever (BHF) was first identified in 1959 as a sporadic hemorrhagic illness in rural areas of Beni department, Bolivia. Clusters of BHF patients were noted the same year, and by 1962 BHF was recognized as a new epidemic infectious disease. In 1963, Machupo virus (a member of the family *Arenaviridae*) was first isolated from patients with acute hemorrhagic fever in San Joaquin, Bolivia (1). Ecologic investigations established the rodent *Calomys callosus*, which is indigenous to the disease-endemic region of northern Bolivia, as the reservoir for Machupo virus (2,3).

Machupo virus infection in *C. callosus* results in asymptomatic infection with shedding of virus in saliva, urine, and feces; 50% of experimentally infected *C. callosus* are chronically viremic and shed virus in their bodily excretions or secretions (2). Although the infectious dose of Machupo virus in humans is unknown, exposed persons may become infected by inhaling virus shed in aerosolized secretions or excretions of infected rodents, by eating food contaminated with rodent excreta, or by direct contact of excreta with abraded skin or oropharyngeal mucous membranes (4). Reports of person-to-person

